# Endothelial Progenitor Cells as Prognostic Markers of Preterm Birth‐Associated Complications

**DOI:** 10.5966/sctm.2016-0085

**Published:** 2016-07-27

**Authors:** Mariane Bertagnolli, Anne Monique Nuyt, Bernard Thébaud, Thuy Mai Luu

**Affiliations:** ^1^ Department of Pediatrics, Sainte‐Justine University Hospital Research Center, University of Montreal, Montreal, Quebec, Canada; ^2^ Department of Pediatrics, Ottawa Hospital Research Institute, University of Ottawa, Ottawa, Ontario, Canada

## Abstract

Preterm birth is associated with alteration of the vascular tree that can result in disease states such as bronchopulmonary dysplasia and retinopathy of prematurity during the neonatal period and emphysema and hypertension in adulthood. Studies have suggested a potential role for endothelial progenitor cells in the pathophysiology of prematurity‐related complications involving blood vessels; however, this knowledge has never been synthesized. We conducted a systematic review of the published data to examine the characteristics of endothelial progenitor cells in relation to preterm birth in humans. Preterm infants compared with term controls displayed similar or increased circulating/cord blood endothelial progenitor cell counts. However, the preterm endothelial progenitor cells were more vulnerable to exogenous factors such as oxidative stress. A reduced number, in particular of endothelial colony‐forming cells, was associated with bronchopulmonary dysplasia. No studies have examined endothelial progenitor cells beyond the neonatal period. These findings could prove useful in the identification of biomarkers for prognostication or therapeutic strategies for vascular‐related diseases in preterm‐born individuals. Stem Cells Translational Medicine *2017;6:7–13*

**Significance Statement**

Endothelial progenitor cells (EPCs) are central for maintaining healthy blood vessels. A way in which EPCs can be altered right from birth, especially after preterm delivery, is now being discovered. Preterm neonates are at risk of diseases marked by abnormal blood vessel development such as bronchopulmonary dysplasia. In adulthood, these individuals are vulnerable to chronic health problems, including hypertension and emphysema, also characterized by impaired blood vessels. Synthesizing the knowledge about the relationship between EPCs and preterm birth will help clarify whether EPCs can be used for the prediction of diseases occurring after prematurity and whether restoring EPC function can be a target for future treatment.

## Introduction

Worldwide, approximately 10% of infants are born prematurely (<37 weeks of gestation). Advances in perinatology have markedly improved the survival of premature infants but many experience significant morbidities. The first generation of extremely preterm survivors (<28 weeks), who are now entering adulthood, manifest cardiovascular disease risk conditions early in life, such as elevated blood pressure, altered myocardial shape and function, and signs of pulmonary obstruction 1 2 3 4. However, the underlying pathophysiological mechanisms are not well established, hindering the development of biomarkers for early identification of disease risk during the neonatal period and beyond and advances in therapeutic interventions.

Recently, endothelial progenitor cells (EPCs) have emerged as a potential biomarker and therapeutic target that could be used to detect and treat medical complications of preterm birth. EPCs play a critical role during vascular repair and regeneration by homing to sites of tissue injury to restore vascular integrity and ensure normal endothelial function 5, 6. These properties are crucial during organogenesis and postnatal development 5. Mounting evidence suggests EPCs are altered by disorders of pregnancy that can be associated with preterm birth such as diabetes and pre‐eclampsia 7, 8. Furthermore, lower numbers of endothelial colony‐forming cells (ECFCs), a subset of EPCs capable of self‐renewal and de novo vessel formation, were also associated with the development of bronchopulmonary dysplasia (BPD) 9, 10. Taken together, EPC impairment could underlie many of the short‐ and long‐term complications associated with preterm birth. However, the effect of gestational age on EPCs remains unclear. The present review synthesized the existing data to examine the impact of preterm birth on EPCs and determine whether EPC impairment is associated with prematurity‐related conditions.

## Materials and Methods

We searched PubMed, MEDLINE, Embase, CINAHL COMPLETE, and EBM reviews for articles published in English from January 1997 (first study on EPCs) to January 21, 2015, using the medical subject terms “preterm birth” OR “low birth weight” AND “endothelial progenitor cells” ( supplemental online Table 1). The reference lists of relevant reports were manually reviewed for additional citations. The first selection of studies based on title and abstract, assessment of full‐text articles for inclusion, and data extraction and quality assessment without blinding to journal or authorship using an adapted version of the Newcastle‐Ottawa Quality Assessment Scale ( supplemental online Table 2) 11 were performed by two independent reviewers (M.B., T.M.L.). We did not assess the quality of exclusively basic science studies, given the lack of validated scales. During the process, all disagreements were settled by consensus between the two reviewers or, on occasion, after discussion with a third party (A.M.N.). We included observational studies conducted on humans born preterm (<37 weeks of gestation) or with a birth weight <2,500 g in which EPCs were characterized by a specific pattern of cell surface markers (i.e., combination of stem/progenitor cell, endothelial cell, and hematopoietic cell) or by in vitro assessment of colony formation. Owing to the heterogeneity of the studies regarding EPC measures, a meta‐analysis was not performed.

## Overview of Published Data

Our systematic review included 18 articles summarized in Tables 1 and 2. All studies measured EPCs in cord and peripheral blood up to 6 months after birth. No study has examined preterm EPCs beyond 6 months or during infancy; therefore, at present, it is unclear whether observed EPC abnormalities persist throughout the lifespan and could contribute to an increased risk of later cardiovascular diseases.

**Table 1:** EPC characterization methods and EPC count/functional assessment

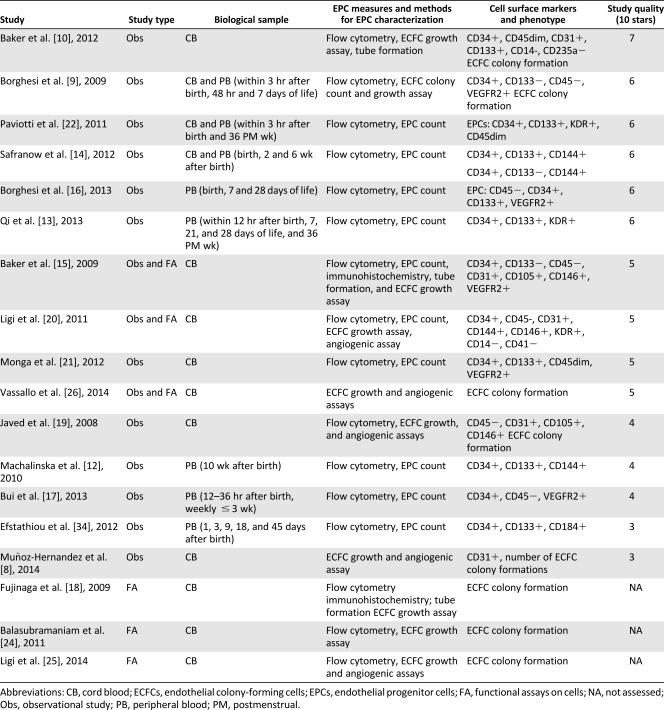

**Table 2:** Results and main findings of studies comparing preterm versus term, preterm‐related complications, and in vitro conditions on EPC numbers and ECFC function

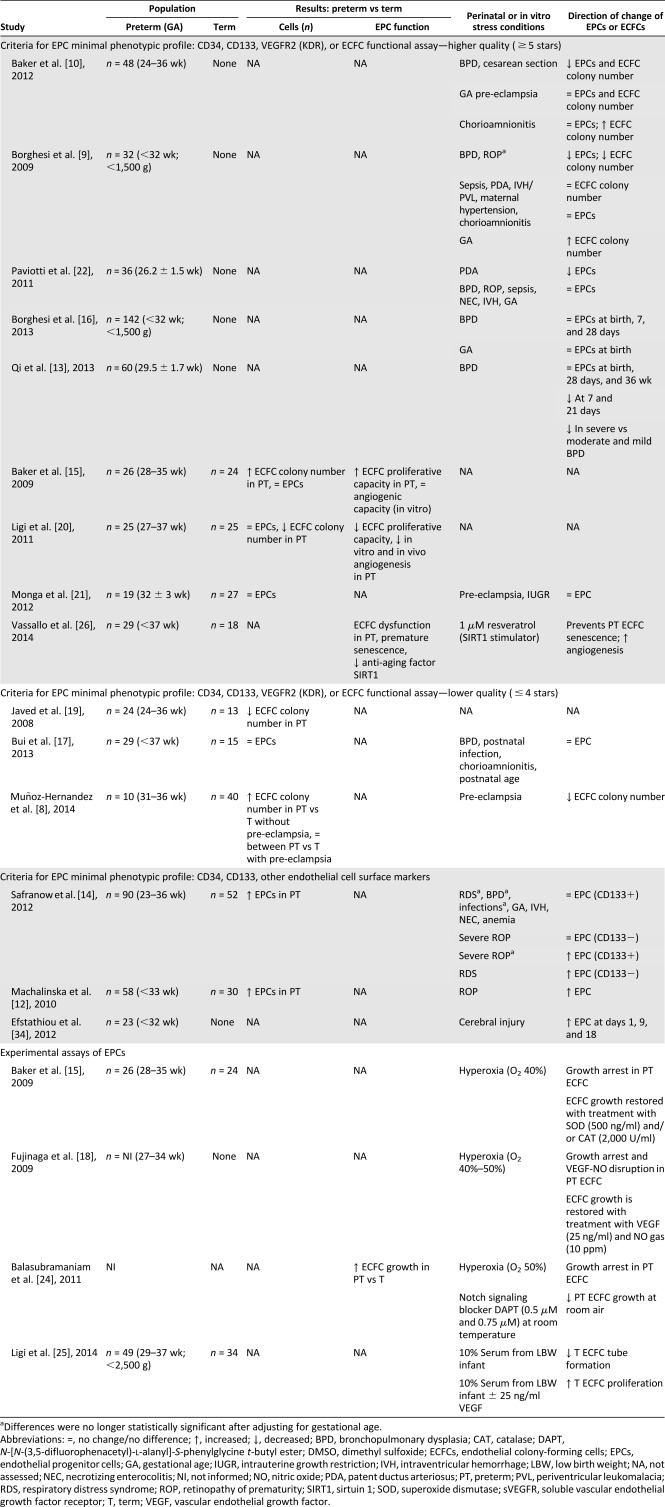

Given the lack of clear consensus regarding EPC definition in the included studies, several methods to characterize these cells were used and included cell enumeration by flow cytometry, number of colony formations in vitro, cell functional assays (in vitro growth and tubular formation), and in vivo vasculogenesis. Most studies searched for a combination of stem cell markers (CD34+, CD133+) and endothelial markers (CD31+, CD105+, CD144+, CD146+, VEGFR2+/KDR+) 9, 12 13 14, with some also assessing the lack of expression of a hematopoietic marker (CD45−) 10, 15 16 17 18 19 20 21 22, which further discriminated EPCs from hematopoietic cells 23. Furthermore, ECFC assays were performed in a subset of studies with functional analysis of cultured cells to assess proliferative (clonogenic assay) and/or angiogenic (capillary tube formation) properties in vitro 9, 10, 15, 19, 20, 24 25 26 27. Three studies characterized endothelial cell phenotype by testing ECFC human vessel‐forming activity in vivo in a murine model 8, 20, 26. Finally, some studies combined several criteria to confirm the nature of the isolated cells as EPC 9, 15, 20, 26.

### Comparison of EPC Count and Function Between Preterm and Term‐Born Infants

Ten studies examined the effect of preterm birth on EPC count and function (Table 2). Two studies found increased EPC counts determined by flow cytometry in preterm versus term infants 12, 14, and four reported no difference at all 15, 17, 20, 21. Four studies enumerated ECFC colonies after cord blood culture with contradictory results. The preterm infants displayed reduced numbers of ECFC colonies in two studies 19, 20 and greater numbers in the remaining two 8, 15.

Safranow et al. 12, 14 sampled preterm and term infants at birth and 2 and 6 weeks later and characterized EPCs using cell surface markers CD34+/CD133+/CD144+ and CD34+/CD133−/CD144+. At birth and 2 weeks, the preterm neonates displayed higher numbers of EPCs than did the term controls; however, the counts were similar at 6 weeks. In 22% of the cohort, the EPC counts were tracked longitudinally and shown to decrease in preterm infants but to remain constant in term controls.

Three studies 15, 17, 21 additionally searched for a lack of CD45 expression in combination with CD34+/VEGFR2+ markers for cell characterization and did not detect any difference in the cord and peripheral blood EPC counts between preterm and term infants. Ligi et al. 20 also performed flow cytometry to count cells using only CD34+/CD45− markers and found no difference between preterm and term infants. However, in their study, although the counts were similar, ECFC function was impaired in the preterm infants. Preterm cord blood grew approximately six times fewer ECFC colonies compared with term controls after 14 days in culture. Preterm ECFCs also displayed reduced proliferative capacity and impaired vessel formation in vitro and in vivo. Whether the observed findings in that study were related to preterm birth per se is unclear given that 27% of preterm infants were born after a hypertensive gestation (known to be associated with increased antiangiogenic factors) compared with 5% of the term controls. The other study, from Javed et al. 19, that revealed lower ECFC colony counts in preterm versus term cord blood did not report on pregnancy complications.

Baker et al. 15 also cultured ECFCs from the umbilical cord blood of 26 preterm and 24 term neonates (presence of maternal hypertension not mentioned). They found that after 14 days of culture, in contrast to the observations by Ligi et al. 20, preterm cord blood grew four times more ECFC colonies than did term blood, owing to the greater proliferation capacity of preterm ECFCs. However, vessel‐forming ability in vitro did not differ between the preterm and term groups. Likewise, Muñoz‐Hernandez et al. 8 observed higher counts of ECFC colonies after 4 weeks of cord blood culture in moderate to late preterm (*n* = 5) versus term (*n* = 30) after normotensive pregnancies.

### Preterm EPC Counts in Association With Preterm‐Related Complications

Eleven studies examined the link between EPC counts and maternal conditions and neonatal complications associated with preterm birth (Table 2). Borghesi et al. studied 142 consecutive preterm neonates <32 weeks’ gestational age or <1,500 g 9, 16. ECFCs (CD34+/CD45−/VEGFR2+/CD133−) were cultured from a subset of 32 preterm cord blood samples and found to be three times lower in those who subsequently developed BPD (O_2_ dependence at 28 days). Moreover, those born at <28 weeks of gestational age had lower ECFC counts than those of the remaining preterm infants born at older gestational ages. Furthermore, infants with retinopathy of prematurity (ROP) displayed reduced numbers of ECFCs, although the difference was no longer statistically significant after adjusting for the degree of prematurity. Other morbidities, including sepsis, patent ductus arteriosus (PDA), brain injury, maternal hypertension, and chorioamnionitis, were not associated with the ECFC counts. In contrast to ECFCs, the same investigators did not observe any correlation between the EPC (CD34+/CD45−/VEGFR2+/CD133+) counts at birth or at 7 or 28 days and any of the studied antenatal or postnatal conditions 16.

Likewise, Paviotti et al. 22 did not find any relationship between the EPC counts at birth and neonatal outcomes, including BPD. However, infants who subsequently developed PDA and required treatment displayed lower EPC counts than did those who did not. The results obtained by Qi et al. 13 somewhat overlapped those of Borghesi et al. 9, 16 and Paviotti et al. 22, with preterm infants with or without O_2_ dependence at 28 days displaying similar numbers of EPC soon after birth. Infants who developed BPD had lower CD34+/CD133+/KDR+ cell counts at 7 and 21 days, but the levels were again comparable at 28 days and 36 weeks.

Baker et al. assembled a cohort of 48 preterm infants for whom cord blood was cultured for ECFC colonies and enumeration was further performed through flow cytometry 10. Infants who developed BPD had reduced ECFC counts compared with those without BPD. In addition, infants born after a diagnosis of clinical chorioamnionitis or after vaginal birth (vs cesarean section) had higher ECFC counts.

Bui et al. provided pilot data to show trends toward lower counts of CD34+/CD45−/VEGFR2+ in peripheral blood over a 3‐week period in infants who were later diagnosed with BPD 17. Infants born to mothers with chorioamnionitis or who developed postnatal infections also tended to mount a response with higher EPC counts; however, their study was underpowered to demonstrate a significant association.

Although Baker et al. 10 and Monga et al. 21 did not detect any statistically significant association between pre‐eclampsia and EPC counts, Muñoz‐Hernandez et al. 8 analyzed a highly selected subgroup of preterm infants and reported a decrease in cord blood ECFC counts after a pre‐eclamptic pregnancy compared with normotensive pregnancy, but the sample size was very low.

Finally, a few studies reported higher EPC counts in preterm infants with specific postnatal complications. Safranow et al. 14 found higher cord blood EPCs in infants with severe ROP, as well as with BPD and sepsis, but the differences were no longer statistically significant after adjustment for gestational age. At 10 weeks, the same researchers observed higher circulating EPC counts in infants with ROP versus without ROP 12.

### ECFC Function and Experimental Conditions Relevant to Preterm Birth

A series of studies have delved further into mechanistic pathways that could explain differences between preterm and term EPCs by investigating in vitro ECFC function in response to prematurity‐related environmental stressors (hyperoxia) and proangiogenic factors. All studies assessing ECFC cells were defined as cobblestone‐shape colonies formed within a range of 5–28 days and kept using similar media conditions. A summary of findings is described in Table 2. First, hyperoxia (O_2_ 40%) was shown to significantly inhibit the growth potential of preterm ECFCs, with minimal effect on term cells 15, 18, 24. Treatment of preterm ECFCs with the antioxidants superoxide dismutase and catalase improved the proliferative properties under hyperoxic stress 15. Hyperoxia‐induced oxidative stress impaired ECFC growth, possibly through inhibition of proangiogenic and proliferative Notch signaling 24 and disruption of the vascular endothelial growth factor (VEGF)‐nitric oxide (NO) pathway as demonstrated by recovery of preterm ECFC proliferation under hyperoxic conditions with VEGF and NO treatment 18. Ligi et al. 25 further illustrated that incubation of cord blood ECFCs with preterm sera—shown to have lower concentrations of VEGF compared with term sera—blunted cell growth and that addition of VEGF restored ECFC proliferation. Reduced proliferative and angiogenic capacity could also result from accelerated stress‐induced senescence of preterm ECFCs 26. Treatment of preterm ECFCs with resveratrol, a SIRT1 (sirtuin 1; anti‐aging factor) stimulator, enhanced both cell growth and vessel‐forming function.

### EPC Vulnerability in Preterm Birth

Overall, EPCs and, in particular, ECFCs were either similar or increased in preterm infants compared with term controls. After a preterm birth, infants are still rapidly growing and developing. This stage corresponds to the third trimester of gestation, a period of substantial microvasculature development and mobilization of stem cells, which are systemically increased in the fetus compared with postnatal levels 28. However, in vitro analyses suggest that preterm ECFCs are more susceptible to oxidative stress (e.g., hyperoxia) compared with term ECFCs, which could be mediated by disruption of proangiogenic pathways, such as VEGF and NO 18, 25, and accelerated cell senescence 26. Taken together, these findings suggest that antenatal and postnatal stressors can significantly affect preterm ECFCs, which are more vulnerable at this stage of development compared with term cells. Altered ECFC function could contribute to the subsequent disease states, notably BPD, observed in preterm infants.

BPD, the most common complication of prematurity, was frequently associated with reduced EPC counts and impaired cell function. Preterm birth occurs at the saccular stage of lung development when the airways and pulmonary vessels come together. Lung angiogenesis, through secretion of VEGF and NO, among others, participates in the subsequent alveolarization process 29. Decreased ECFC levels and function might hinder pulmonary vascular development and repair, thus increasing the risk of later BPD 30, 31.

ROP is another complication characterized by uncontrolled vascular growth into the vitreous mediated by hypoxia, inflammation, and oxidative stress, which induce angiogenic factors (e.g., VEGF) 32, 33. These pathophysiological processes can be reconciled with the observation of reduced cord blood EPCs 9 and increased peripheral EPCs at 10 weeks in infants who develop ROP 12. However, the association between EPCs and ROP is less documented than that with BPD.

## Conclusion

At birth, circulating EPCs, including the ECFC subtype, are present, most often at similar or sometimes increased numbers, in preterm‐born neonates compared with term controls. However, in vitro cell analysis indicated increased vulnerability of preterm ECFCs to hyperoxia‐induced oxidative stress with resulting dysfunction. Finally, convincing evidence supports the relationship between reduced numbers of the EPC subtype ECFC and the development of BPD but not with other relevant perinatal complications for now.

Given the burden of preterm birth complications at the individual and societal level, unraveling the mechanisms underlying alterations in preterm EPCs could pave the way for new treatment options that restore EPC function. However, careful cell characterization that also includes functional assays to define EPC is of upmost importance.

## Author Contributions

M.B.: conception and design, collection and/or assembly of data, data analysis and interpretation, manuscript writing, final approval of manuscript; A.M.N.: financial support, manuscript writing, final approval of manuscript; B.T.: manuscript writing, final approval of manuscript; T.M.L. conception and design, financial support, collection and/or assembly of data, data analysis and interpretation, manuscript writing, final approval of manuscript.

## Disclosure of Potential Conflicts of Interest

T.M.L. has received research funding from the Merck, Sharpe, and Dohme grant program–University of Montreal Faculty of Medicine. The other authors indicated no potential conflicts of interest.

## Supporting information

Supporting Information
