# Supplementary material for: Endothelial Progenitor Cells as Prognostic Markers of Preterm Birth‐Associated Complications
Source: Stem Cells Transl Med. 2016 Jul 27;6(1):7–13. doi: 10.5966/sctm.2016-0085 (PMC5442749; doi:10.5966/sctm.2016-0085)
Supplement: Supplementary file 1 — Supporting Information [file SCT3-6-007-s001.pdf]

RUNNING TITLE: ENDOTHELIAL PROGENITOR CELLS AND PREMATURITY

PERSPECTIVES

ENDOTHELIAL PROGENITOR CELLS AS PROGNOSTIC MARKERS OF PRETERM BIRTH-ASSOCIATED COMPLICATIONS: A SYSTEMATIC REVIEW

Mariane Bertagnolli PhD[1]; Anne Monique Nuyt MD[1]; Bernard Thébaud MD, PhD[2]; Thuy Mai Luu MD[1]

[1] Department of Pediatrics, Sainte-Justine University Hospital Research Center, Université de Montréal, Montreal, Canada

[2] Department of Pediatrics, Ottawa Hospital Research Institute, University of Ottawa, Ottawa, Canada

SUPPLEMENTAL MATERIAL

Supplemental Material Table S1 - Full search strategy

Supplemental Material Table S2 - Adapted Newcastle-Ottawa Quality Assessment Scale

Supplemental Material: Table S1 - Full search strategy

1	Preterm birth (unlimited)	((preterm*[TIAB] OR pre-term*[TIAB] OR micropreterm*[TIAB] OR prematur*[TIAB] OR pre-matur*[TIAB] OR preemie*[TIAB] OR low birth weight[TIAB] OR lowbirth weight[TIAB] OR lbw*[TIAB] OR vlbw*[TIAB] OR elbw*[TIAB] OR term-equivalent age[TIAB] OR small for date[TIAB]) OR ((neonat*[TIAB] OR neo-nat*[TIAB] OR newborn*[TIAB] OR new-born*[TIAB] OR infant*[TIAB] OR baby[TIAB] OR babies[TIAB] OR gestational*[TIAB]) AND (underweight[TIAB] OR under-weight[TIAB] OR SGA[TIAB] OR small for[TIAB]))) OR ((preterm*[OT] OR pre-term*[OT] OR micropreterm*[OT] OR prematur*[OT] OR pre-matur*[OT] OR preemie*[OT] OR low birth weight[OT] OR lowbirth weight[OT] OR lbw*[OT] OR vlbw*[OT] OR elbw*[OT] OR term-equivalent age[OT] OR small for date[OT]) OR ((neonat*[OT] OR neo-nat*[OT] OR newborn*[OT] OR new-born*[OT] OR infant*[OT] OR baby[OT] OR babies[OT] OR gestational*[OT]) AND (underweight[OT] OR under-weight[OT] OR SGA[OT]

		OR small for[OT])))
2	Preterm birth (controlled)	Infant, Premature[MH] OR Infant, Extremely Premature[MH] OR Premature Birth[MH] OR Infant, Low Birth Weight[MH] OR Infant, Very Low Birth Weight[MH] OR Infant, Extremely Low Birth Weight[MH] OR Infant, Small for Gestational Age[MH]
3		#1 OR #2
4	endothelial progenitor cells (unlimited)	(endotheli*[TIAB] AND (progenitor*[TIAB] OR stem[TIAB] OR colony-forming[TIAB])) OR (endotheli*[OT] AND (progenitor*[OT] OR stem[OT] OR colony-forming[OT]))
5	endothelial progenitor cells (controlled)	Endothelial Progenitor Cells[MH] OR Endothelial Cells[MH:NOEXP]
6		#4 OR #5
7		(#3 AND #6) AND (1994:2016[DP] AND (english[LA] OR french[LA]))

Supplemental Material: Table S2 - Adapted Newcastle-Ottawa Quality Assessment Scale

A study can be awarded a maximum of one star for each numbered item within the Selection categories. A maximum of two stars can be given for Comparability. A maximum of three stars can be given for the first category for Outcome and one star for the second category for Outcome.
**A) SELECTION (Maximum of 4 stars)**<br><br>**A1) Cohort study design**<br><br>1) Representativeness of the exposed cohort<br>a) truly representative of the average preterm population*<br>b) somewhat representative of the average preterm population in the community*<br>c) selected group of users<br>d) no description of the derivation of the cohort<br><br>2) Selection of the non-exposed cohort<br>a) drawn from the same community as the exposed cohort*<br>b) drawn from a different source<br>c) no description of the derivation of the non-exposed cohort<br><br>3) Ascertainment of preterm status<br>a) secure record (e.g. birth records)*<br>b) written self-report

c) not mentioned

4) Demonstration that outcome of interest was not present at the beginning of the study

a) yes*

b) no or not mentioned

A2) Case-control design

1) Is the case definition adequate?

a) yes, with independent validation*

b) yes, e.g. record linkage or based on self-reports

c) no description

2) Representativeness of the cases

a) consecutive or obviously representative series of cases*

b) potential for selection biases or not stated

3) Selection of controls

a) controls are derived from the same cohort*

b) controls are derived from a different cohort

c) no description

4) Definition of controls

a) no history of disease*

b) no description of source

B) COMPARABILITY (Maximum of 2 stars)

1) Comparability of cohorts on the basis of analysis

a) study controls for age (EPC analysis performed at the same chronological time)*

b) study controls for sex*

C) OUTCOME (Maximum 4 stars)

1) Assessment of outcome (Maximum 3 stars)

1a) Were EPCs identified correctly?

a) right cells selected (CD34+, VEGFR2+ and CD45- assessed by flow cytometry)*

b) cells grown in culture to look at colony-forming capacity*

c) assessment of angiogenic capacity (in vitro or in vivo)*

d) unconventional technique

e) no description at all

1b) Was technician blinded to neonatal history?

a) yes*

b) no
